# Editorial: Neuromodulatory Function in Auditory Processing

**DOI:** 10.3389/fncir.2022.898646

**Published:** 2022-05-17

**Authors:** R. Michael Burger, Conny Kopp-Scheinpflug

**Affiliations:** ^1^Department of Biological Sciences, Lehigh University, Bethlehem, PA, United States; ^2^Division of Neurobiology, Department Biology II, Ludwig Maximilian University, Munich, Germany

**Keywords:** neuromodulation, inferior colliculus, superior olive, auditory cortex, acetylcholine, auditory

Neuromodulatory systems are generally known to play roles in complex behaviors such as the sleep/wake cycle, attention, arousal and learning (Picciotto et al., 2012). Canonical modulatory circuitry emanates from discrete nuclei throughout the brain, and projects broadly to virtually every region. However, most scientific investigation of modulatory signaling has focused on higher order computational centers in the forebrain, where its impact on neural response properties may be understood in the context of complex functions such as attention or arousal (McGinley et al., 2015; Carcea et al., 2017). Much less is understood about how neuromodulation shapes processing in subcortical sensory regions, or how it may influence circuitry during development.

The works presented here provide a broader view of how neuromodulation may influence auditory processing; from brainstem to cortex, from computationally simple regions to the complex, and on time scales ranging from transient to long-term. The challenges of investigating neuromodulation in vertebrates stem from poor understanding of the conditions under which modulatory ligands are released, the wide variety of receptors that bind each of them, and the spatial and temporal ranges of action which is so different from classical neurotransmitters. In tackling these challenges in the vertebrate brain, the auditory system provides the experimental advantages of a robust functional understanding of auditory processing from the ear to cortex.

## New Modulatory Pathways

This topic's contents include what are likely to be enduring discoveries providing new insights into the anatomy of cholinergic circuitry influencing auditory nuclei. Beebe et al. focused on cholinergic input to the superior olive, a cluster of nuclei that processes several fundamental aspects of auditory input, and Noftz et al. documented cholinergic inputs to the inferior colliculus (IC) in unprecedented detail using viral vectors, tract tracing, and immunohistochemistry. Together they describe major output projections of cholinergic neurons in the pontomesencephalic tegmentum (PMT), which is not just a primary source of cholinergic output in the brain, but may serve as a hub for multiple neuromodulators.

## Modulation Over Short Time Scales

Several works appearing in this volume illustrate the power of investigating neuromodulatory physiology in the auditory system, where modulation of stimulus driven activity in real-time can be interpreted in its functional context. Instkerveli and Metherate demonstrated how activation of nicotinic signaling increases response gain and shortens response latency across populations of auditory cortex neurons. These changes improved response reliability across trials and countered adaptation to repeated stimuli which may be important for processing ongoing signals including speech. Similarly, Rivera-Perez et al. revealed the cellular mechanisms for nicotinic gain control in a genetically defined population of neurons in the IC. Specifically, they showed that α_3_β_4_ nicotinic receptors are primarily responsible for mediating a depolarizing inward current that both boosts membrane excitability and enhances summation of excitatory inputs. Together these papers show that modulation by acetylcholine can rapidly and consequentially enhance responses to sound in two major auditory centers.

Auditory modulation is not limited to cholinergic input however, and several studies collectively demonstrated the wide variety of factors that are brought to bear on auditory computation. Serotonin is a neurotransmitter well known to be released in specific behavioral contexts such as mating. Polese et al. presented anesthetized mice with broadband vocalizations while recording IC neuron responses in the presence or absence of serotonin agonists. They showed that serotonin released in the context of mating has the potential to sharpen neural selectivity for specific vocalizations and call features.

Two review contributions highlight bodies of work that reveal complex and varied influences of metabotropic glutamate receptors (mGluRs) and nitric oxide (NO) signaling. Goel et al. illustrate the many ways that mGluRs control both presynaptic release at excitatory synapses and simultaneously modulate the excitability of the postsynaptic cells in the sound localization circuitry of both birds and mammals. Kopp-Scheinpflug and Forsythe review how NO, synthesized in response to Ca^++^ entry during synaptic activity, can mediate a myriad of effects in both postsynaptic and presynaptic neurons. Importantly, NO as a soluble messenger has the potential to act on neurons away from its site of production and independently of synaptic connections, including non-auditory or multisensory neurons.

## Modulators May Shape Circuitry Over Long Time Periods

Modulation may not necessarily derive from discrete clusters of functionally similar neurons releasing a canonical transmitter, nor is modulatory function temporally constrained to influence processing in time scales limited to stimulus duration or behavioral state. One example of modulatory release from principal auditory neurons themselves is provided by Wollet and Kim. Activity-dependent BDNF release is known to influence synaptic plasticity during long term potentiation Xu et al. (2010). Here, Wollet and Kim demonstrate that sound driven BDNF signaling also influences auditory circuitry on developmental time scales. Heterozygous BDNF (+/–) mutant mice failed to develop normal frequency-dependent patterning of intrinsic neural properties. A second compelling example is presented by Pagella et al., who showed that Urocortin 3, a neuropeptide transmitter released during stress, and its receptor CRFR2, are expressed broadly in the auditory pathway by principal and interneurons, respectively, suggesting a reverse modulation from principal neurons to the canonical modulatory neurons. Urocortin 3 knockout animals have been shown to be particularly sensitive to noise damage suggesting that the auditory pathways express modulators that confer an auto-protective function (Fischl et al., 2019). Finally, Knipper et al. propose a model by which trauma induced hyperexcitability along the auditory pathway influences BDNF signaling to disrupt the balance of excitation and inhibition. They go on to propose that downstream changes to NO signaling may disrupt the neural-vascular interface which may cause deficits beyond the auditory system.

These studies open new lines of inquiry beyond investigations of excitation and inhibition in principal auditory neurons to include the additional layers of complexity provided by modulation from the ear to cortex and back again. Further, it is evident that modulatory circuitry shapes neural responses on time scales ranging from long-lasting developmental processes to immediate effects in the mature organisms. We hope the work presented here will stimulate further exploration into what promises to be a rich and emerging field of auditory neuroscience.

## Author Contributions

All authors listed have made a substantial, direct, and intellectual contribution to the work and approved it for publication.

## Conflict of Interest

The authors declare that the research was conducted in the absence of any commercial or financial relationships that could be construed as a potential conflict of interest.

## Publisher's Note

All claims expressed in this article are solely those of the authors and do not necessarily represent those of their affiliated organizations, or those of the publisher, the editors and the reviewers. Any product that may be evaluated in this article, or claim that may be made by its manufacturer, is not guaranteed or endorsed by the publisher.
